# On-Chip Detection of the Biomarkers for Neurodegenerative Diseases: Technologies and Prospects

**DOI:** 10.3390/mi11070629

**Published:** 2020-06-28

**Authors:** Chao Song, Suya Que, Lucas Heimer, Long Que

**Affiliations:** 1 Electrical and Computer Engineering Department, Iowa State University, Ames, IA 50011, USA; csong@iastate.edu (C.S.); ljheimer@iastate.edu (L.H.); 2 Ames High School, Ames, IA 50010, USA; 889264que@ames.k12.ia.us

**Keywords:** neurodegenerative diseases, on-chip detection, point-of-care diagnostics

## Abstract

Alzheimer’s disease (AD), Parkinson’s disease (PD) and glaucoma are all regarded as neurodegenerative diseases (neuro-DDs) because these diseases are highly related to the degeneration loss of functions and death of neurons with aging. The conventional diagnostic methods such as neuroimaging for these diseases are not only expensive but also time-consuming, resulting in significant financial burdens for patients and public health challenge for nations around the world. Hence early detection of neuro-DDs in a cost-effective and rapid manner is critically needed. For the past decades, some chip-based detection technologies have been developed to address this challenge, showing great potential in achieving point-of-care (POC) diagnostics of neuro-DDs. In this review, chip-based detection of neuro-DDs’ biomarkers enabled by different transducing mechanisms is evaluated.

## 1. Introduction

Worldwide, the elderly population is growing. Within the next 30 years, 30 percent or more of the population will be aged 60 or more all over the world. In the US, the percentage of people age 65 or more is expected to reach 20 percent by 2030 [1]. Elderly people are more likely to be affected by diseases such as glaucoma, hypertension, Alzheimer’s disease (AD), Parkinson’s disease (PD), Huntington’s disease (HD), cardiac disease and cancers. Of these, glaucoma, AD, PD and HD can be classified as neurodegenerative diseases (neuro-DDs) due to degeneration, loss of functions and death of neurons with aging. Currently, these diseases are usually incurable, leading to the progressive loss of structure or function of neurons, and eventually the death of neurons. Diagnosis and treatment of these diseases at their early stages are critical to improve the quality of life for the elderly [2,3,4,5,6,7]. The ultimate goal of diagnosing preclinical neuro-DDs is to be able to apply some interventions at their early stages, which may postpone, mitigate or even avoid the upcoming neurological impairment that would occur if the conditions are left unattended. However, current clinic diagnostic methods for neuro-DDs are expensive and time-consuming, requiring skilled personnel to operate some sophisticated equipment. Hence, it has been becoming increasingly and critically important to develop simple-to-use, inexpensive, convenient yet reliable diagnostic methods such as chip-based point-of-care (POC) diagnostics.

POC diagnostic methods are simple medical tests that can be carried out at the bedside or source-limited settings. Toward this goal, chip-based sensing module is one of the key elements since it can potentially facilitate the monolithically fabrication of the sample preparation module, the signal reading module, the data analysis module and the data transmission module on a single chip. Currently, while a variety of chip-based sensing platforms have been developed, but in many cases, the reading of transducing signals still requires off-chip instruments, not to mention the data analysis and transmission. For instance, a fluorescence microscope is needed for measuring fluorescent signals from chip-based fluorescence sensors, and an atomic force microscope (AFM), as a standard technique for monitoring micro-cantilever deflection, is required for monitoring the displacement or the resonant frequency of the MEMS cantilever sensors. Clearly, both instruments cannot be readily miniaturized or made portable, resulting in a challenge for their POC applications. Hence, there are still quite a few technical challenges ahead to achieve chip-sensing based POC diagnostics for neuro-DDs. It should be also noted that due to the advances of microelectronics and photonics, it has been becoming increasingly promising to co-fabricate the signal reading elements on the sensor chip [8,9].

### 1.1. Neuro-DDs and Major Diagnosis Methods

#### 1.1.1. Alzheimer’s Disease

AD, a chronic progressive neurodegenerative disease, is the most prevalent type of dementia. Its most common early symptom is short-term memory loss. As the disease progresses, symptoms include problems with language, disorientation (including the likelihood of getting lost), mood swings, loss of motivation, lack of managing self-care and behavior issues. The AD mechanisms have been well established, the main features of the disease are dysfunction of neurons, loss of connections between neurons in close proximity, and subsequent death of neurons [10,11,12]. Neuron death is attributed mainly to the deposition of Aβ42 protein fibril aggregates onto neurons [13]. Three forms of Aβ42 protein exist in human cerebrospinal fluid (CSF): monomer, oligomer and fibrillar in composition. The monomer form of Aβ42 is dissolved in the CSF, circulating in the system. The oligomer Aβ42 is the bonding form or crosslinked form of monomer Aβ42, which can also be dissolved in CSF and can potentially be crosslinked, forming insoluble fibrillar Aβ42. The insoluble fibrillar Aβ42 deposited onto neurons can disrupt connections between adjacent neurons. Once these connections are lost, neurons die, forming large areas of plaque in the brain. After formation of oligomers and fibrillar Aβ42, the concentration of monomer Aβ42 in CSF is thus decreased. Another phenomenon of AD is the significantly increased concentration of the T-tau biomarker in CSF and blood [14,15]. T-tau consists of proteins that can stabilize microtubules, existing mainly in neural cells of the central nervous system. T-tau, like Aβ42, is considered toxic to neurons. The tau hypothesis proposes that excessive or abnormal phosphorylation of tau in CSF may result in the transformation of normal adult tau into the paired helical filament-tau and neurofibrillary tangles (NFT) [16]. The accumulation of hyperphosphorylated tau in neurons is believed to lead to neurofibrillary degeneration [17]. Such tangles are toxic to cells, resulting in cell death and cognitive decline. Tau can also be toxic to neurons by accumulation inside the cells, through a process involving enzyme stimulated phosphorylation of tau. For the past decades, Aβ42, T-tau and phosphorylated tau (P-tau) have been widely regarded as the promising clinic biomarkers for AD [18,19,20]. Clinically relevant levels of Aβ42 and T-tau in CSF are 382.2 ± 102.0 pg/mL and at least >300 pg/mL, respectively [12,14].

Current diagnostic methods for AD are based on neuron imaging including magnetic resonance imaging (MRI), positron emission tomography (PET) and single photon emission computed tomography (SPECT) [21]. They are expensive and time-consuming for diagnosing AD. In addition, the challenges for these methods are for increased sensitivity and specificity in the diagnosis of AD. Hence, new sensitive, inexpensive and rapid diagnostics methods are urgently needed, particularly in the early stages of AD or before symptoms occur.

#### 1.1.2. Parkinson’s Disease

PD is another chronic progressive neurodegenerative disease, which is the second most common neurodegenerative disorder, affecting 1–2% of the population over the age of 65 years [22]. The loss of dopaminergic neurons in the brain region, which is known as substantia nigra (SN), usually results in bradykinesia, rigidity, tremor and postural instability in the patients [23]. One of the pathological features of PD is 50–70% neuronal loss in the SN region. Neuronal inclusions include α-synuclein protein, which are located both in the neuronal cell body (Lewy bodies) and neurites (Lewy neurites) [24]. It has been proposed that oligomers of α-synuclein are the toxic species, which cause the neuronal death in the early stages of PD [25]. Increasing evidence has shown that dysfunctional regulation and misfolding of α-synuclein in Lewy bodies involve in the pathogenesis of PD [25,26]. The monomeric and the aggregated forms of α-synuclein proteins are found in CSF and serum. Formation of plaques due to the aggregation of the α-synuclein causes degeneration of neurons, making it a promising biomarker for early diagnosis and prognosis monitoring of PD [27]. Clearly routine test of α-synuclein in the blood/serum is a more realistic than the test of α-synuclein in CSF for diagnosing PD since collecting CSF requires an invasive procedure. However, α-synuclein levels in the blood from PD patients and healthy individuals are diverse and even contradictory to each other. Specifically, while the clinical relevant levels of α-synuclein in the blood of healthy people is in a range from 77 to 12 ng/mL [28,29], but the elevated levels of α-synuclein from PD patients have been reported by some research [28,30], the decreased levels of α-synuclein from PD patients by other research [31]. More efforts need to be devoted to resolving or explaining the contradict observations.

PD is usually diagnosed based on medical history, a review of signs and symptoms, and a neurological and physical examination of the patients. Patients may be also examined by a specific single-photon emission computerized tomography SPECT scan called a dopamine transporter (DAT) scan [32]. The detection of the promising biomarker such as α-synuclein in serum or CSF may provide a complementary diagnosis of PD to improve the diagnostic accuracy. Toward this goal, chip-based detection including paper-based lateral flow assay [33] is an ideal option.

#### 1.1.3. Glaucoma

Glaucoma is one type of eye disease that results in damage to the optic nerve and vision loss due to high ocular pressure in the eye. Recent research has shown that glaucoma is also a kind of neuron degenerative disease [34,35,36]. The disease, which affects about 2 million people in the U.S., occurs more commonly among older people. Screening for glaucoma is usually performed as part of a standard eye examination by an optometrist. Testing for glaucoma should include measurements of the intraocular pressure (IOP) via tonometry, anterior chamber angle examination and examination of the optic nerve for any visible damage. A formal visual field test should be performed. The retinal nerve fiber layer can be assessed with imaging techniques such as optical coherence tomography, scanning laser polarimetry and scanning laser ophthalmoscopy.

Current glaucoma screening techniques including IOP measurement have poor sensitivity and are ineffective for early diagnosis of primary glaucoma (PG) [37,38]. Given these limited screening methods, there is a great need for new biomarkers of early PG. Among many possible biomarkers [39,40], of a particular interest is the role of cytokines, which is involved in oxidative stress and inflammation [41]. However, in order to measure, screen and validate the biomarkers, tears have to be obtained from patients and then analyzed using a high-sensitivity enzyme-linked immunosorbent assay (ELISA) and mass spectrometry (MS) [42,43]. These widely used methods for tear-biomarker analysis are expensive, time-consuming and require the collection of tears from the patient. Given the limited amount of tears, the procedure of tear-sampling may be challenging and inconvenient to the patient. Hence, sensors that can monitor the biomarkers in tears in situ are very attractive.

In this review, we will only focus on the chip-based detection methods based on MEMS and nanotechnologies [44,45,46,47] for monitoring some promising protein biomarkers of AD, PD and glaucoma disease.

## 2. Sensors for Neuro-DDs Biomarker Detection

### 2.1. Review of Some Chip-Based Sensing Technologies

**Figure 1:**
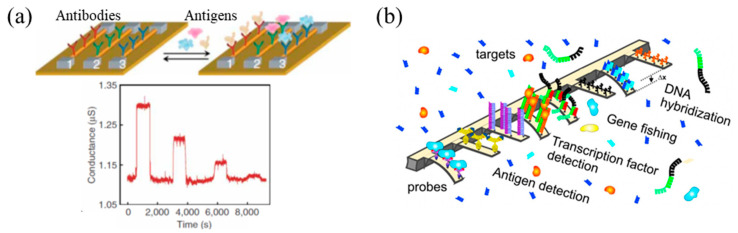
Two non-optical representative sensing technical platforms: (**a**) nano-electronics- nanowires (NWs) and (**b**) micro/nano-mechanics-cantilevers. Reproduced from reference [49] with permission from Springer Nature.

Over the past decades, numerous efforts have been undertaken to develop highly sensitive and selective sensors for medical diagnosis, disease monitoring, drug discovery, detection of environmental pollutants and biological agents [48]. Among them, three categories of sensors in terms of transducing mechanisms have been widely used. The transducing mechanisms include electrical, mechanical and optical responses. For instance, electrical transduction is enabled by carbon nanotubes (CNTs) or silicon nanowires (NWs) where the electrical conductance of the CNTs or silicon NWs is modulated upon the binding between the receptors (antibodies-Abs) immobilized on them and the probes (antigens-Ags; Figure 1a) [49,50]. Mechanical transduction is achieved by MEMS cantilevers (Figure 1b) [51,52] where the binding between antigens to an antibody-immobilized cantilever surface changes the cantilever’s surface stress, resulting in its bending and resonant frequency shifting.

**Figure 2:**
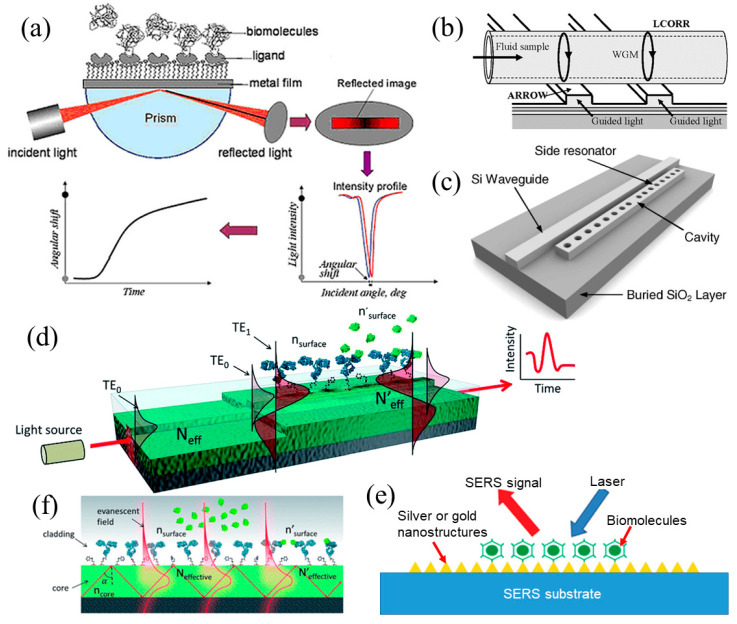
(**a**) A light is beamed upon a metal film through a prism and the reflected beam image shows a dark line due to surface plasmon resonance (SPR). The intensity profile of the reflected beam exhibits a dip or minimal intensity at the resonance angle. A SPR experiment measures the position shift of the dip (the angle shift) upon molecular adsorption, and this shift represents the adsorption kinetics when plotted as a function of time; (**b**) liquid core ring resonator (LCORR) sensor; (**c**) photonic crystal-based biosensor; (**d**) bimodal waveguide interferometric (BiMWI) biosensor; (**e**) evanescent field biosensor and (f) surface enhanced Raman spectroscopy (SERS) sensor. Reproduced from references [61,62,66,71] with permission from AIP Publishing, Optical Society of America and Royal Society of Chemistry.

Optical sensing techniques are based on various sensing mechanisms including chemiluminescence, fluorescence, light absorption and scattering, reflectance, interference, surface plasmon resonance (SPR) and Raman scattering. Of these, fluorescence is currently the major detection technique in bioscience, chemistry, life science, pharmaceutical and medical research. However, attaching extrinsic tags such as fluorophores or quantum dots to biomolecules for large scale studies can often be tedious, expensive and not easily generalizable. Furthermore, this labeling process may perturb or even change their properties. This is particularly relevant when studying properties of proteins [48,53,54,55]. Subtle changes in the binding affinities and associated kinetics of protein molecules, either by addition of an extrinsic tag or through tag-induced conformational changes in protein molecules, can have a profound influence on some functions of protein molecules. One example is the study of the recognition of stereo-chemically modified double-stranded DNA (dsDNA) by specialized proteins in a living system using fluorescence-based sensing [56]. Thus the tools for real-time label-free detection, as an emerging technique requiring further development, are becoming increasingly attractive since no significant sample preparation such as the attachment of fluorophores or quantum dots to the sample, is needed. The major label-free techniques by optical means are propagation surface plasmon resonance (SPR; Figure 2a) [57], Raman spectroscopy [58,59], localized SPR (L-SPR) [60], liquid core ring resonator technology (Figure 2b) [61], photonic crystal nanostructures (Figure 2c) [53,62,63] and interferometer-enabled biosensors including Mach–Zehnder interferometric (MZI) biosensor [64], bimodal waveguide interferometric (BiMW) biosensor (Figure 2d) [8,65], Young interferometer (YI) biosensor [66,67], Fabry−Pérot interferometric (FPI) biosensor [68] and the nanopore thin film-based reflectometric interference spectroscopic (RIfS) sensors [69,70,71,72].

For all aforementioned label-free optical biosensors, they can also be categorized based on their specific optical characteristics of transduction: (1) Evanescent-wave biosensors (Figure 2e), in which the transducing signals are achieved by the interactions between the biomolecules and the evanescent wave on the sensing surface, include optical resonators (liquid core ring resonators (LCORR), photonic crystal, etc.), interferometer-enabled biosensors (MZI sensors, FPI sensors, BiMW sensors, YI sensors and RIfS biosensors), SPR sensors, L-SPR sensors, etc. (2) Light scattering biosensors, in which the transducing signals are generated due to the electromagnetic (EM) scattering by the biomolecules and nanoscale structures on the sensing surface, include Raman spectroscopy sensors, SERS sensors (Figure 2f), etc.

A SPR sensor shown in Figure 2a is usually used in the laboratory environment to detect biological and chemical species. Surface plasmon (SP) can be excited at a metal and dielectric interface by a monochromatic or near-monochromatic optical source. The fields associated with the SPR extend into the medium adjacent to the interface and decay exponentially away from it. Penetration into the medium is in the range of <200 nm [57]. Consequently, the SP is very sensitive to the changes in the environment near the interface, and therefore is used as a sensing probe. Upon the excitation of the SPR, a valley in reflectance from the interface occurs. The position of the valley shifts to a different angle of incidence if there are any changes in the local environment at the interface. Raman spectroscopy is a powerful tool for analyzing the chemical composition of materials [73]. It offers information about the material’s electronic and vibrational structure and its distinctive chemical fingerprint for different materials, making it especially attractive for ultra-selective analysis. The surface enhanced Raman spectroscopy (SERS) effect was discovered in the 1970s [74]. The Raman signal of pyridine dramatically increases when absorbed on a roughened Ag electrode (or SERS substrate). The high loss of optical power is a big challenge for maintaining the high sensitivity. Liquid core ring resonators (LCORR) technology (Figure 2b) was demonstrated for multiplexed biosensing by placing LCORR in contact with multiple antiresonant reflecting optical waveguides (ARROWs) [61]. It utilizes the ARROWs to excite the whispering gallery modes of a LCORR sensor. A photonic crystal-based biosensing device is illustrated in Figure 2c [62]. It consists of a silicon waveguide with a 1D photonic crystal microcavity (side resonator) that is adjacent to the waveguide, which is evanescently coupled to each other. A change in the refractive index of the near field region surrounding the optical cavity results in a shift of the resonant wavelength.

In the following sections, some representative sensors based on optical, electrical or mechanical transducing signals for detecting biomarkers of AD, PD and glaucoma are detailed.

### 2.2. AD Biomarker Detection

**Figure 3:**
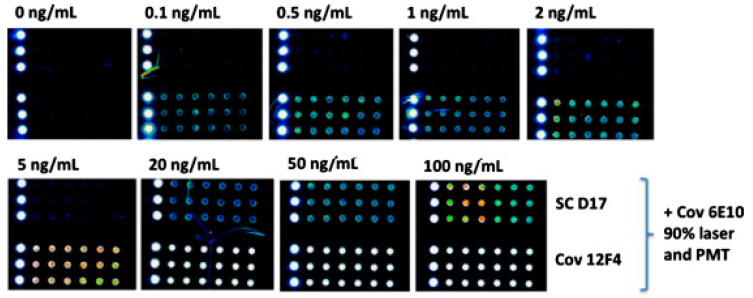
Fluorescence images for detecting Aβ42 with concentrations from 0 to 100 ng/mL using SC-D17 (upper array) or Cov-12F4 (lower array) as the capture antibody and Cov-6E10 as the detection antibody. Reproduced from reference [75] with permission from Elsevier.

Silicon/silicon oxide (Si/SiO_2_) based sensor: This chip (Figure 3) is prepared by depositing a layer of SiO_2_ on a silicon wafer [75]. When the thickness of SiO_2_ layer is properly selected (100 nm for this chip), the emission of any fluorophore of choice can potentially be enhanced due to the constructive interference, resulting in significant improvements in detection sensitivity. Briefly, to detect Aβ1–42 and Aβ1–39, the capturing antibodies against Aβ are firstly immobilized on the chip. The chip is then blocked with 50 mM ethanolamine solution in 1 M TRIS/HCl pH 9 for 1 h to mitigate the non-specific binding, followed by rigorous rinsing with water and drying under a stream of nitrogen. Aβ1–42 or Aβ1–39 diluted in artificial cerebrospinal fluid (CSF) is applied on the chip for incubation. Thereafter, the chip was rigorously washed with washing buffer (0.05 M Tris/HCl pH 9, 0.25 M NaCl, 0.05% *v/v* Tween 20) for 10 min with stirring and then rinsed with water. Biotin-labeled secondary antibody at 1 µg/mL in PBS was applied on the chip for 1 h incubation. After being washed with PBS and water for 10 min each, the Cyanine 3 labeled with streptavidin at 2 µg/mL in PBS was applied and incubated for 1 h, followed by another round of rinsing with PBS and water for 10 min each. Finally, the fluorescence images and intensities were obtained by a ProScanArray scanner (PerkinElmer, Boston, MA, USA). It is anticipated that all these steps can be significantly simplified, potentially made automatically and thus more user-friendly by integrating this chip with a microfluidic interface [76].

Some representative measurements are shown in Figure 3. In these experiments, in order to identify the capturing antibodies to achieve the highest signal intensity and specificity for Aβ42 and Aβ39, SC-D17, NT-11H3, NT-8G7, Cov-4G8 and Cov-12F4 are tested as the capturing antibodies. It has been found that antibodies SC-D17 and Cov-12F4 offer higher signal intensity and specificity for Aβ42. Using these two antibodies as capturing antibodies, the fluorescence results of arrays for the detection of 0, 0.1, 0.5, 1, 2, 5, 20, 50 and 100 ng/mL of Aβ42 in ACSF after 2 h of dynamic incubation are shown in Figure 3. It has been found that the optimum conditions for detecting Aβ42 are to use Cov-12F4/Cov-6E10 matched antibody pair and 2 h of dynamic incubation. Due to the fluorescence signals enhanced by the constructive interference effect of the silicon/silicon oxide thin film, the limit-of-detection (LOD) of Aβ42 in ACSF was improved to 73.07 pg/mL.

**Figure 4:**
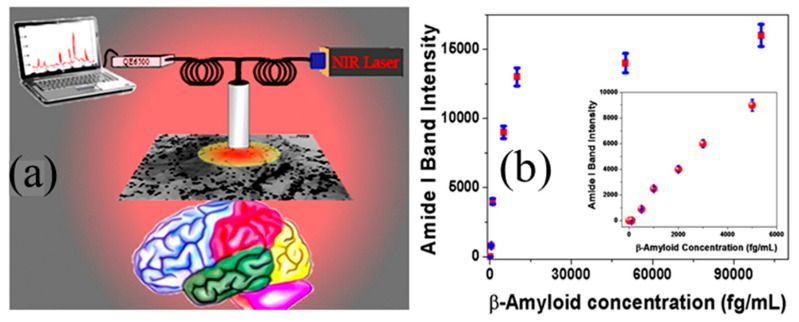
(**a**) Schematic of plasmonic-magnetic hybrid graphene oxide-enabled surface-enhanced Raman spectroscopy (SERS) platform for detecting AD biomarkers and (**b**) measured SERS amide I band intensity from Aβ conjugated nanoplatform changes with concentration between 0 and 6 pg/mL. The limit-of-detection (LOD) can be as low as 500 fg/mL. Reproduced from reference [80] with permission from American Chemical Society.

SERS enabled sensor: surface-enhanced Raman spectroscopy (SERS) was adopted to measure Aβ42 and T-tau dissolved in CSF [77,78,79]. One type of the SERS chip was enabled by core-shell nanoparticle attached 2D hybrid graphene oxide based multifunctional nanoplatform (Figure 4) [80]. It was found that the core-shell nanoparticle was very effective in enhancing Raman signal by generating electromagnetic field hot spot. Graphene oxide can chemically enhance the Raman signal by influencing the aromatic molecule interaction with a large surface area.

Results (Figure 4b) show that much lower concentrations can be detected by this type of SERS chip than with the ELISA kit (0.312 ng/mL for Aβ42, 0.15 ng/mL for T-tau). It has been demonstrated as low as 100 fg/mL for both Aβ42 and T-tau can be detected.

**Figure 5:**
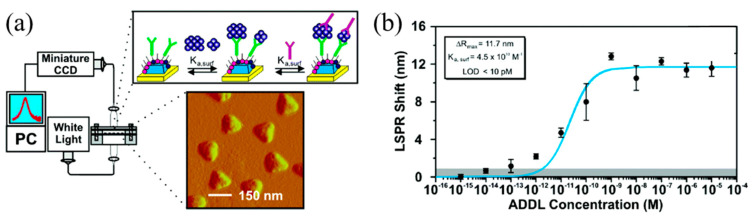
(**a**) Sketch and experimental setup for localized surface plasmon resonance (LSPR) based sensors and (**b**) some experimental results of amyloid-*β* derived diffusible ligands (ADDLs) detection. Reproduced from reference [81] with permission from American Chemical Society.

Optical LSPR biosensor: localized surface plasmon resonance (LSPR) has also been utilized to detect amyloid-*β* derived diffusible ligands (ADDLs). The LSPR nanosensor (Figure 5a) is fabricated by nanosphere lithography [81]. Usually LSPR leads to extraordinary light absorption or reflection at specific wavelengths. As a result, the extraordinary light absorption or reflection caused a light intensity peak or dip in the broadband spectrum. The peak or dip in the spectrum was highly related to the refractive index, shape, size and environmental conditions of the substrate material. UV-vis extinction measurements from the sensor were collected by an optical fiber coupled to a spectrometer (Ocean Optics, Dunedin, FL, USA; Figure 5a).

Using a sandwich assay, this type of nanosensor can quantitatively determine the concentration of ADDL (Figure 5b), providing a unique method for analyzing the aggregation mechanism of this putative AD pathogen at physiologically relevant monomer concentrations. Experiments found that the binding constants of two ADDL epitopes to the specific anti-ADDL antibodies were 7.3 × 10^12^ M^−1^ and 9.5 × 10^8^ M^−1^, respectively. The LOD of this type of LSPR sensor for detecting ADDL was conservatively estimated to be 10 pM.

**Figure 6:**
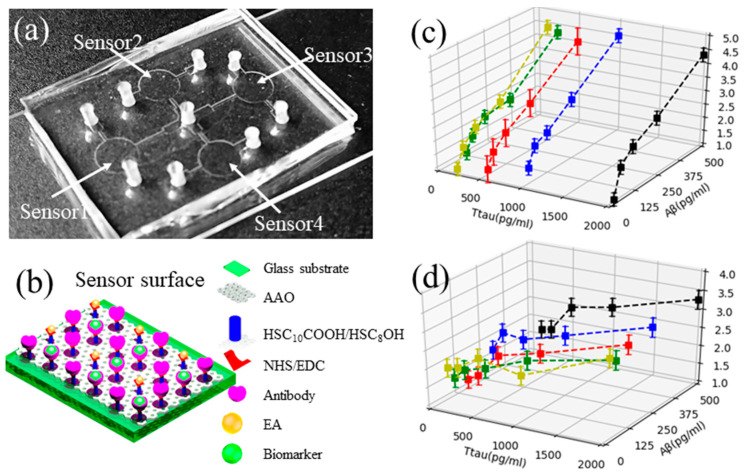
(**a**) Photo of fabricated sensors on chip for detecting Aβ42 and T-tau; (**b**) sketch showing the functionalized sensor surface for detecting biomarkers; (**c**) measured transducing signals of Aβ42 in Aβ42-T-tau mixture using an Aβ42-sensor and (**d**) measured transducing signals of T-tau in Aβ420T-tau-mixture using a T-tau-sensor. Reproduced from reference [84] with permission from Elsevier.

Arrayed nanopore-based sensor: using the unique optical property of the nanopore thin film-based RIfS sensor for label-free biodetection [82,83], a chip consisting of arrayed sensors fabricated from the anodic aluminum oxide (AAO) nanopore thin film on a glass substrate has been developed as shown in Figure 6a [84]. The Aβ42 antibody or T-tau antibody were immobilized on the chemically functionalized sensing surface (Figure 6b). Upon the binding of the biomarker to the antibody, the reflected optical signal (interference fringes) from the sensor will shift. The shift of the optical signal was used as the transducing signal. These sensors on the chip were used as a reference for control experiments, for detection of Aβ42, T-tau and the mixture of Aβ42 and T-tau, respectively.

The detection of Aβ42 and T-tau in both buffer and in CSF for AD was carried out. Typical detection time was less than 20 min after a biomarker was applied on the functionalized nanosensor. The procedure for the detection was much faster than neuroimaging methods such as magnetic resonant imaging (MRI). It was found that as low as 7.8 pg/mL of Aβ42 in buffer and 15.6 pg/mL of T-tau in buffer can be readily detected with high specificity and very good repeatability. Furthermore, the detection of both Aβ42 and T-tau spiked into CSF samples was demonstrated as shown in Figure 6c,d. Based on these measurements, the feasibility to monitor these biomarkers in clinical samples was demonstrated using the sensors. It is anticipated that the detection of clinic CSF samples can be readily carried out when available after the cellular components in the samples are removed.

**Figure 7:**
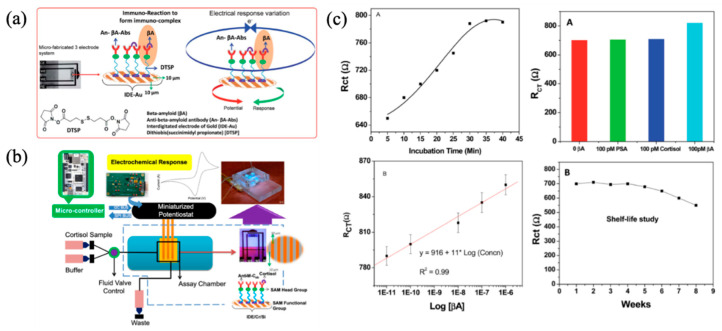
(**a**) Principle of interdigitated electrode of the gold (IDE-Au) immunosensor; (**b**) the system with IDE-Au based immunosensor and (**c**) measured results of Aβ using this system and the stability of the IDE-Au based immunosensor. Reproduced from reference [85] with permission from The Royal Society of Chemistry.

An interdigitated electrode of the gold (IDE-Au) sensor: An electrochemical immunosensor for detecting Aβ42 was developed. The fabricated IDE-Au sensor is shown in Figure 7a,b [85]. First, the Aβ42 antibody was immobilized onto the sensor surface, which was modified by a dithiobissuccinimidyl propionate self-assembled monolayer (DTSP-SAM), followed by applying Aβ42 on the sensor. Second, the electrochemical impedance spectroscopy (EIS) was carried out to monitor the impedance change during the bonding process of Aβ42 antibody and Aβ42. Based on the EIS measurements, the developed IDE-Au immunosensor had a high sensitivity of 11 kΩ/M with a regression coefficient of 0.99 as shown in Figure 7c. In addition, this sensor was selective with a limit of detection (LOD) of 10 pM and had a detection range varying from 10 pM to 100 nM. The shelf-life of the IDE-Au immunosensor was also evaluated. It was found that the sensor was stable for 30 days at 4 °C, while the magnitude of the electrochemical response reduced rapidly after 30 days (Figure 7c).

**Figure 8:**
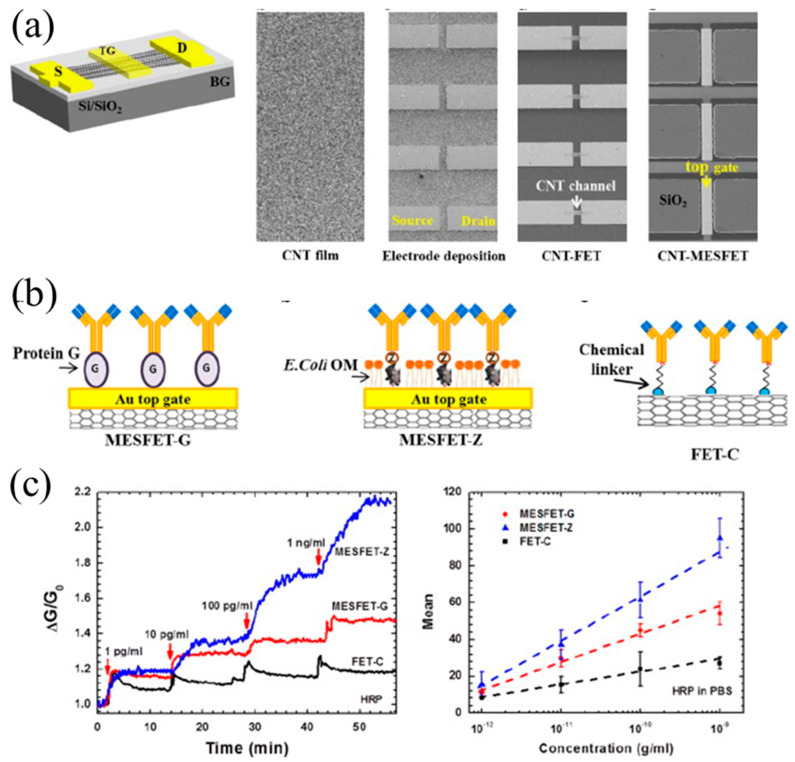
(**a**) Sketch of metal–semiconductor field-effect transistor (MESFET) based sensor and optical micrographs of the carbon nanotube (CNT) film and the procedure to obtain the sensors; (**b**) sketch showing the surface functionalization process and (**c**) measured results of Aβ biomarker with MESFET sensor. Reproduced from reference [86] with permission from Elsevier.

Metal–semiconductor field-effect transistor (MESFET) based biosensor: a semiconducting carbon nanotube (CNT) film-based biosensor with a metal semiconductor field effect transistor structure (CNT-MESFET) has been developed for detecting Aβ as shown Figure 8a [86]. In this type of senor, an Au strip was fabricated on the middle of the CNT film channel, resulting in a Schottky barrier forming at an interface between the Au strip and the CNT.

To detect Aβ42, the Aβ antibody was first immobilized on the Au top gate of the CNT-MESFET biosensor (Figure 8b). When the chemical linker/Aβ antibody binds with Aβ42, the threshold voltage of the CNT-MESFET is changed. As a result, the conductance of channel was modulated and so was the source-to-drain current of the CNT-MESFET (Figure 8c). Hence, the concentration of Aβ42 could be determined by measuring its source-to-drain current. A linear relationship was observed in the plot of conductance change versus Aβ42 concentration on the semi-logarithm scale for the range of 10^−12^–10^−9^ g/mL. It was demonstrated that as low as 1 pg/mL Aβ42 could be readily detected in human serum using the CNT-MESFET biosensor.

### 2.3. PD Biomarker Detection

**Figure 9:**
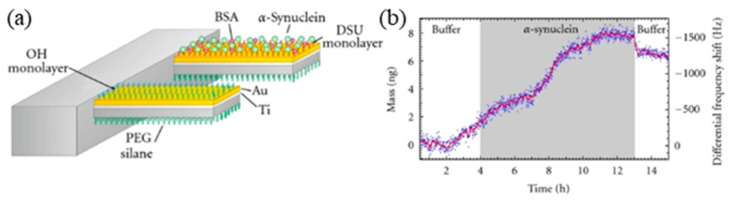
(**a**) Schematic of the functionalized MEMS cantilever for detecting *α*-synuclein and (**b**) bound mass on the surface of the cantilever and frequency of the cantilever versus time. The left axis shows the bound mass on the cantilever, the right axis shows the corresponding differential frequency shift. The grey area indicates the period that 10 µg/mL *α*-synuclein in 20 mM sodium phosphate buffer is flowing through the fluidic chamber at a rate of 3.3 µL/min. Reproduced from reference [90] with permission from Hindawi Publishing Corporation.

MEMS cantilever detection of PD biomarkers: a variety of biomolecules have been detected using the MEMS cantilever device [87,88,89]. This type of MEMS cantilever device has also been adapted for detecting *α*-synuclein [90]. A functionalized cantilever for detecting *α*-synuclein and a reference cantilever are schematically shown in Figure 9a. Silicon cantilever arrays (IBM Research Laboratory, Switzerland) were used in the experiments. Each cantilever within an array was 500 µm long, 100 µm wide and 1 µm thick (with a 10 nm tolerance of the thickness). The arrayed cantilevers had a pitch of 250 µm. The use of an array of cantilevers allowed multiple tests and in situ references in one single experiment, thereby resulting in increased throughput experiments.

Using the MEMS cantilevers, the aggregation of the protein *α*-synuclein can be detected in a quantitative, label-free manner. Specifically, a test cantilever was functionalized with the dithobis(succinimidyl undecanoate) (DSU) monolayer on top side and PEG saline on back side (Figure 9a). The DSU monolayer provides the binding sites for the *α*-synuclein in solution. While the reference cantilever was functionalized with the OH monolayer on the top side and PEG saline on the back side (Figure 9a). Then the functionalized cantilever was operated in a dynamic mode in the presence of *α*-synuclein monomers in solution. The response of the test cantilever due to any nonspecific adsorption of protein to the either side (PEG back side or OH terminated top side) of the cantilever could be canceled out by the response from the reference cantilever. As shown in Figure 9b, it was found that approximately 6 ng of *α*-synuclein was aggregated on the surface of the cantilever over a 9-h period, and a small amount (1 ng) of *α*-synuclein was removed from the surface by following monomers buffer through the cantilever, which were close to conventional fluorescence measurements of *α*-synuclein aggregation under similar conditions. In addition, this detection method requires a concentration of *α*-synuclein protein 50 times smaller than that of the fluorescence method and potentially offers a faster response time.

**Figure 10:**
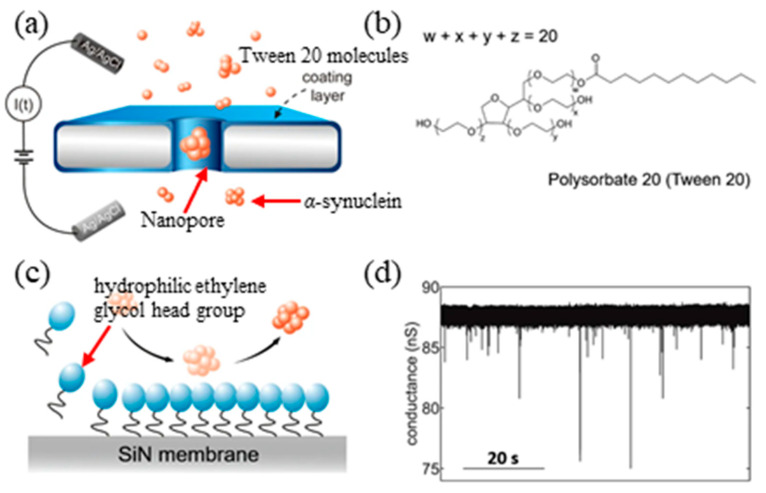
(**a**) Sketch of the experimental setup: the flow cell is separated by silicon nitride membrane with a nanopore. The silicon nitride membrane is coated by a layer of tween 20 molecules to mitigate non-specific binding; (**b**) the chemical structure of tween 20; (**c**) sketch showing the assemble process of tween 20 on hydrophobic silicon nitride membrane and the compact coating layer reduce irreversible non-specific adsorption of *α*-synuclein oligomers and (**d**) measured current traces of nanopore under 100 mV with *α*-synuclein sample incubated for 96 h at pH 9. Reproduced from reference [94] with permission from Springer Nature.

Single nanopore for detecting PD biomarkers: solid-state single nanopore has been extensively used for DNA sequencing and biomolecule detection by monitoring the changes of ionic current or conductance of the single nanopore for the past years [91,92,93]. Detection of *α*-synuclein using solid-state nanopore has also been demonstrated [94,95]. A schematic diagram of *α*-synuclein protein detection using a single nanopore is shown in Figure 10a. A flow cell was separated by a 40 nm thick silicon nitride membrane with a 20 nm nanopore, which was formed using a focused electron beam in TEM. Translocation of *α*-synuclein proteins across the nanopore was achieved by applying an electric field, resulting in characteristic current blockade on the ionic current trace. The current signal through the nanopore was recorded by two Ag/AgCl electrodes connected to the patch-clamp amplifier by immersing the two electrodes in two reservoirs of the flow cell filled with an electrolyte solution (1 M KCl, 10 mM HEPES, pH 9).

In order to mitigate/avoid non-specific adsorption between *α*-synuclein and nanopore surface so that continuous detection of *α*-synuclein translocation through the nanopores can be realized, a tween 20 (Figure 10b,c) coating method on the SiN nanopores was developed. The time-dependent kinetics of *α*-synuclein oligomerization was studied using the solid-state nanopores (Figure 10d). In the experiments, it was found that four types of oligomers were formed during aggregation under controlled incubation condition by analyzing the translocation of *α*-synuclein incubated over a range of times. In addition, the effect of lipid small unilamellar vesicles (SUVs) on *α*-synuclein oligomerization process has been investigated, indicating that the dramatic enhancement of the aggregation rate of *α*-synuclein results from the presence of 20% 1,2-dioleoyl-sn-glycero-3-[phospho-l-serine] (DOPS) [96,97]. All these results suggest that solid-state nanopores are a suitable platform to investigate heterogeneous and vital oligomerization of *α*-synuclein in situ, thereby providing important insights of its aggregation process, which is regarded as the key role in the pathogenesis of PD.

**Figure 11:**
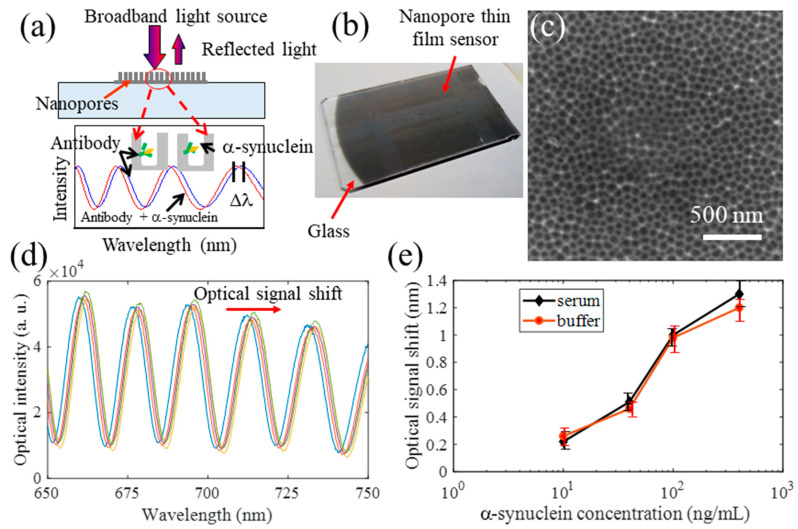
(**a**) Schematic of the nanopore thin film sensor on glass; (**b**) photo of a nanopore thin film sensor fabricated on glass; (**c**) SEM images of the nanopores in the sensor; (**d**) representative measured optical signals (i.e., interference fringe shift) with increased concentrations of *α*-synuclein in buffer and (**e**) measured optical signals of *α*-synuclein in buffer and spiked in serum.

Nanopore thin film sensor: Recently the nanopore thin film-based RIfS sensor fabricated on a glass substrate have been developed to detect *α*-synuclein in buffer and serum in our lab [98]. The illustration of the sensor and its operational principle is shown in Figure 11a. A photo of a fabricated nanopore thin film sensor on glass is given in Figure 11b. A SEM image of the nanopores in the thin-film sensor is shown in Figure 11c. The fabrication process is similar to that reported previously [99]. The sensor surface is functionalized using EDC/NHS chemistry so that the antibody for *α*-synuclein can be immobilized on the surface, followed by applying ethanolamine (EA) on the sensor in order to mitigate or avoid non-specific binding.

For all the measurements, after applying the samples of *α*-synuclein, the thin film sensors are placed on a stirring plate with an incubation time of 30 min. Then the sensors were rinsed rigorously with buffer to remove unbounded chemicals. One representative measurement in Figure 11d shows the shift of optical signal from the sensor when different concentrations of *α*-synuclein in buffer were detected. The measured optical signal of *α*-synuclein in buffer is shown in Figure 11e, clearly the shift of the optical signal increased with its concentration. The measurements of *α*-synuclein spiked in serum were also carried out as displayed in Figure 11e. As shown, the measured signals in buffer and in serum were very consistent, indicating the sensors had very good specificity. In addition, it was found that *α*-synuclein of 10 ng/mL could be readily detected using this type of sensors without any optimization. For specificity evaluation, two closely related proteins, Aβ42 and T-tau, were tested. It was found the optical signals for Aβ42 and T-tau were significantly smaller than those of *α*-synuclein of the same concentrations, indicating its high specificity of the nanopore thin film sensor for detecting *α*-synuclein.

**Figure 12:**
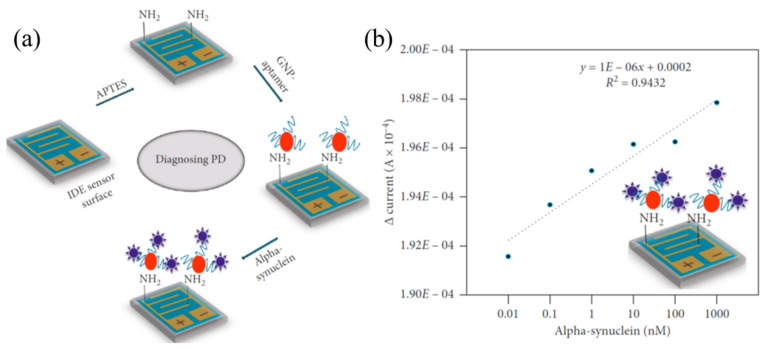
(**a**) Schematic illustration of the surface functionalization steps of the IDE sensor for detecting α-synuclein. First, the surface is modified by APTES to immobilize the GNP-aptamer, then alpha-synuclein is detected. (**b**) The measured responses of α-synuclein of different concentrations, and the linear regression analysis. The limit of detection is 10 pM. Reproduced from reference [100] with permission from Hindawi Publishing Corporation.

Interdigitated electrode (IDE) aptamer sensor: silver IDE electrodes fabricated on silicon wafer have been developed to detect α-synuclein [100]. Different from other sensors, this IDE sensor uses aptamer as the probe to detect α-synuclein at a low level on an amine-modified IDE sensing surface. A schematic illustration of the IDE sensor for detecting α-synuclein by an aptamer-based probe is shown in Figure 12a. The procedure involves the following steps. First, an IDE was functionalized with (3-aminopropyl) triethoxysilane (APTES). Second, the aptamer-gold nanoparticles (aptamer-GNP) conjugates were immobilized on the surface of the functionalized IDE. Thereafter, α-synuclein was detected by the reaction with aptamer. Note that in order to mitigate or avoid the non-specific binding, the concentration of the aptamer needs to be optimized to cover the entire surface of a GNP. After identifying the optimal concentration of aptamer, the aptamer-GNP probe was prepared and consequently immobilized on the surface of an amine-modified IDE.

The limit of detection (LOD) of α-synuclein of the IDE sensor was evaluated by applying different concentrations of α-synuclein from 10 pM to 1 µM. In Figure 12b, the linear regression analysis with a series of different concentrations of α-synuclein and a constant level of aptamer was displayed. Based on the regression analysis and a 3*σ* calculation, it was found that the LOD of the IDE sensor was 10 pM. Specific detection of α-synuclein was performed on the IDE sensor. For this analysis, two proteins, amyloid-beta and tau, were independently tested. It was found only α-synuclein caused a current change, while the other control proteins did not show any significant changes in current from the baseline level, indicating that α-synuclein could be specifically detected by the sensor without any fouling effects.

**Figure 13:**
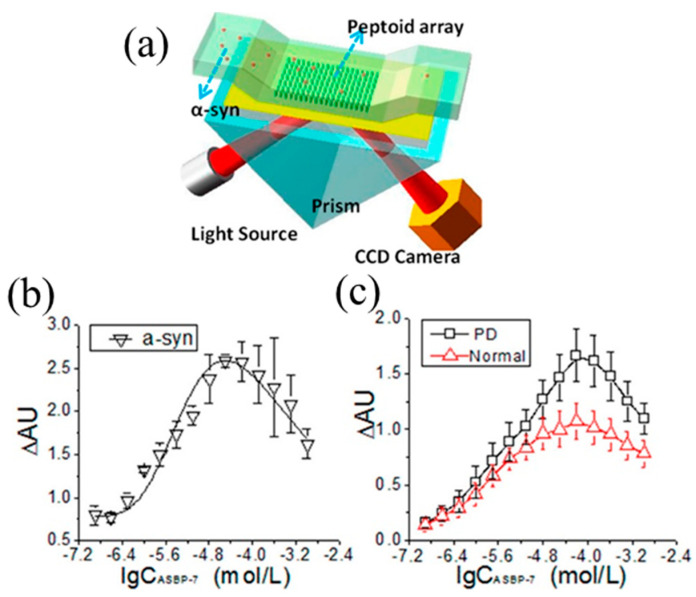
(**a**) Schematic illustration of surface plasmon resonance imaging (SPRi) for screening the peptoid library. (**b**) Measured results for pure α-synuclein binds to different concentrations of ASBP-7. (**c**) Measured results for Parkinson’s disease (PD) serum and for normal serum for healthy people, respectively. Clearly, the measurements of PD serum are differentiated from the normal ones using ASBP-7 at a concentration range from 32 to 512 µM. Error bars represent the standard deviation (*n* = 3 in (**b**), and *n* = 11 in (**c**)). Reproduced from reference [103] with permission from American Chemical Society.

SPR sensor for α-synuclein detection: both SPR and LSPR-based sensors have been utilized for detecting *α*-synuclein [101,102]. As mentioned in Section 2.1, SPR imaging (SPRi) is a label free biosensing platform widely used for studying biomolecular interactions. SPRi has also been adapted to screen for the peptoid with high affinity and specificity to α-synuclein through a combinatorial peptoid library (Figure 13a) [103]. In general, α-synuclein of a series of concentrations is injected into the flow chamber to detect its binding to the peptoids, which is detected by CCD camera, resulting in the changes of the reflected light. Based on these measurements, the binding affinity was determined by analyzing the kinetic interaction between the peptoids to α-synuclein.

Specifically, using SPRi, peptoid α-synuclein binding peptoid-7 (ASBP-7) was identified as a probe that has high affinity and specificity to α-synuclein. Toward this goal, ASBP-7 of different concentrations ranging from 0.125 μM to 1 mM was fixed on the bare gold chip via covalent interaction, and the pure α-synuclein of 1.316 μM was then injected into the flow chamber. As shown in Figure 13b, the highest binding was achieved when the concentration of ASBP-7 was 64 μM. ASBP-7 was then used to detect PD serum. In these experiments, the serum samples from both PD patients and age-matched normal individuals were used. As shown in Figure 13c, a similar trend of the binding signal to that of the pure α-synuclein was observed, namely the binding signal increased first and then declined with the increase of the concentration of ASBP-7. When the concentration of ASBP-7 was between 32 to 512 μM, the binding signals from PD serum was significantly higher than those from normal serum (>1.0 ΔAU). Hence, with ASBP-7 in this appropriate concentration range (32–512 µM), PD could be distinguished from the control by a SPRi platform.

### 2.4. Glaucoma (GA) Biomarker Detection

Biomarker detection in the tear film can assist the diagnosis of early GA, which can potentially provide a complementary diagnosing method to the widely used IOP measurement method for more accurate diagnosis. To date, there are no known highly reliable liquid biomarkers for glaucoma diagnosis, but many potential promising ones have been identified [41,42,43]. One possible biomarker is cytokine Interleukin 12 (IL-12p70) since recent studies have found that the mean concentrations of IL-12p70 in tear film were significantly lower for the diagnosed primary open-angle glaucoma (POAG) group compared to the control group (3.94 ± 2.19 pg/mL in control vs. 2.31 ± 1.156 pg/mL in POAG; *p* = 0.035) [21]. This indicates that measuring the concentration of IL12p70 in the tear film can aid with the diagnosis of early glaucoma, in addition to IOP measurement. As aforementioned, while there are many possible protein biomarkers in tears for glaucoma, there are very few chip-based detection methods to detect them that have been reported. In this regard, soft contact lens based sensors are a particularly suitable platform for in situ detection of biomarkers in tears [104]. There are some contact lens sensors that have been reported, but only to monitor the glucose in tears for diagnosing diabetes [105].

**Figure 14:**
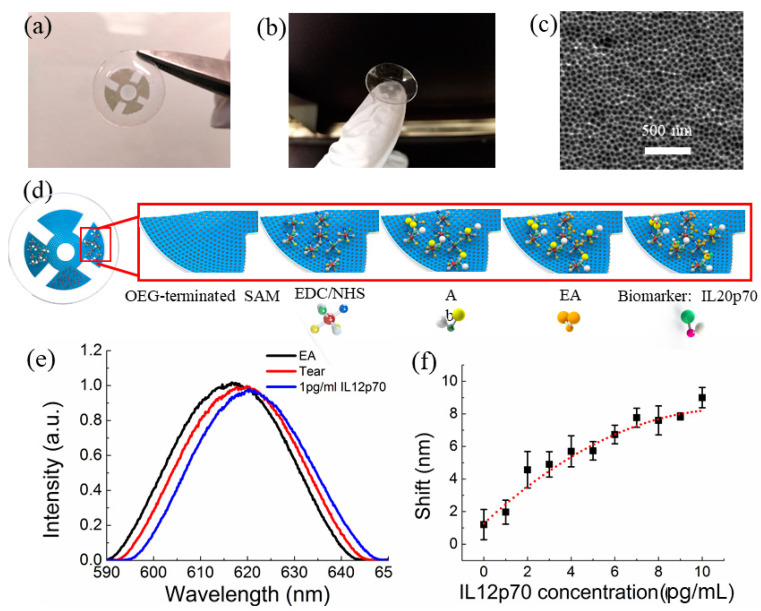
(**a**,**b**) Contact lens with embedded sensors for detection of glaucoma biomarkers; (**c**) SEM images showing the nanopore in the sensors; (**d**) surface functionalization of the sensors on contact lens; (**e**) representative measured optical signals from a sensor on the contact lens with artificial tear and 1 pg/mL IL 12p70 spiked in artificial tear and (**f**) measured optical signals from the sensor with increased concentrations of IL 12p70 in artificial tear. Reproduced from reference [106] with permission from IEEE.

Recently a contact lens sensor (Figure 14a–c) for detecting IL-12p70 has been developed [106]. The biomarker is monitored by measuring the optical reflection signal from the nanopore thin film sensor embedded in the contact lens. The binding of the biomarker with the functionalized surface of the sensor results in a change of the optical path difference (OPD), and thus the shift of the reflected optical signal (interference fringes) from the sensor. The higher the concentration of the biomarker, the larger the shift of the optical signal.

For the measurements, the surface of the contact lens sensor needs to be functionalized with the human IL-12p70 antibody first. The detailed procedure is illustrated in Figure 14d. Briefly, the contact lens sensor surface coated with 10 nm Au is functionalized with human IL-12p70 antibody through 1-ethyl-3-(3-dimethylaminopropyl) carbodiimide (EDC)/*N*-hydroxysulfosuccinimide (NHS) chemistry. This is followed by applying 100 μL 1 M ethanolamine (EA) to block the unoccupied HSC_10_COOH/HSC_8_OH sites activated by the EDC/NHS. Finally, the sensor surface is rinsed with the PBS buffer to remove non-specifically adsorbed proteins. At this stage, the sensor is ready for measuring the biomarker IL-12p70. Mouse IL-12p70 diluted in artificial tears (TheraTears, Akron, Lake Forest, IL, USA) with a concentration ranging from 0 to 10 pg/mL was applied onto the sensor surface for 2 h incubation in sequence, and the shifts of the reflected light were collected. As shown in Figure 14e,f, by increasing the concentration of IL-12p70 from 0 to 10 pg/mL, the shift increased from 1.2 to 9 nm. This indicates that the sensitivity of the contact lens sensor was 0.78 nm/(pg/mL) for IL-12p70 detection. Note that the shift of the artificial tears (0 pg/mL of IL-12p70) was 1.2 nm, indicating the possible non-specific binding of the minerals in the artificial tears with the antibodies of IL-12p70, which can be nulled by subtracting the shift due to the artificial tear. The same principle can be applied for other biomarkers by functionalizing different types of antibodies or aptamers on the sensor surface. Hence, the potential glaucoma biomarker in tears could be screened and validated using this contact lens sensor. As a demonstration, neural cells have been cultured on AAO nanopore thin film on polydimethylsiloxane (PDMS) [107]. It has been found that these cells can grow (spread and divide) normally, indicating its good biocompatibility. It is anticipated that the irritation caused by the contact lens can be totally eliminated by using hydroxyethyl methacrylate (HEMA) to replace PDMS for the contact lenses. The fabrication process of contact lenses using HEMA, a copolymer widely used for soft contact lenses [108], is similar to that using PDMS.

As aforementioned, to our knowledge, very few chip-based sensing platforms have been developed to monitor biomarkers in tears for glaucoma [109]. However, some reported chip-based sensors including contact lens-based sensor or sensing methods, which have been used to monitor other chemicals or biochemicals (such as lysozyme) in tears, can be potentially modified or adapted to detect GA biomarkers. Note that some solution-based sensors utilizing functionalized gold nanoparticles to detect biomarkers in tears have also been reported [110], which are beyond the scope of this review. In the following, a couple of chip-based sensing platforms are described.

**Figure 15:**
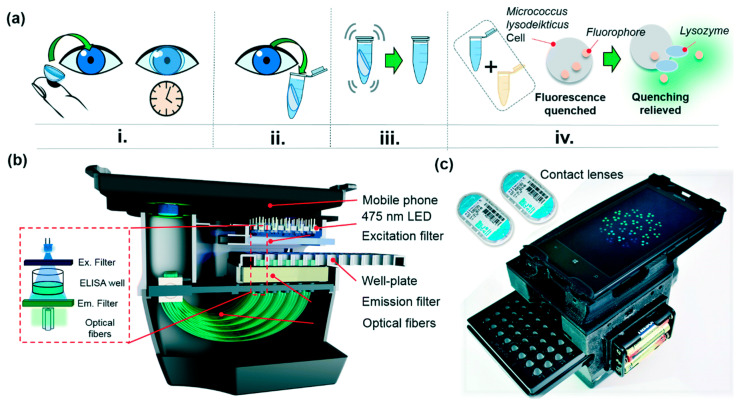
(**a**) (i)–(iv) Sketch: commercial contact lenses are worn for 15 min and then are removed and placed in a collection tube filled with a reaction buffer. The contact lenses are washed in the reaction tube and then are removed and discarded. Of the washed solution 100 μL is mixed with 50 μL of the fluorescent *Micrococcus lysodeikticus* cell solution in an ELISA well and monitored. (**b**,**c**) The schematic illustration of a mobile-phone based well-plate reader and a picture of some contact lens cases with the well-plate reader. Reproduced from reference [111] with permission from The Royal Society of Chemistry.

Contact lens-based lysozyme detection: Using commercial contact lenses (CLs) to collect tear samples, lysozyme levels in tears is subsequently analyzed by a cost-effective and field-portable reader [111]. The procedure to collect tear samples is illustrated in Figure 15a. After CLs are worn for 15 min, they are taken off and immediately placed into several capped 1.5 mL collection tubes. These tubes contain 600 μL of the assay reaction buffer (0.1 M sodium phosphate, 0.1 M NaCl, pH 7.5, containing 2 mM sodium azide as a preservative). After the CLs and reaction buffer solution are mixed thoroughly, the CLs are removed from the tubes. The remaining solution is then analyzed by a mobile-phone based well-plate reader (Figure 15b,c) and image processing.

Specifically, utilizing the CL-based mobile sensing approach, the lysozyme levels of a group of healthy participants over a two-week period were monitored. It was found that the lysozyme levels increased from 6.89 ± 2.02 to 10.72 ± 3.22 μg mL^−1^ (mean ± SD) for CL wearers when they played a mobile-phone game during the wear-duration, inducing an instance of digital eye-strain. In addition, a lysozyme level of 2.43 ± 1.66 μg mL^−1^ in a patient cohort with dry eye disease (DED) in comparison with that of 6.89 ± 2.02 μg mL^−1^ of healthy human participants has been observed. This effort suggests that monitoring the chemicals inside tears can be achieved by a simple tear collection method enabled by CLs, followed by a rapid, easy-to-use and cost-effective measurement system. This system can be potentially adapted to detect the GA biomarkers.

Eyeglasses-based tear biosensing system: recently non-invasive measurements of tear biomarkers by integrating a microfluidic electrochemical detector into an eyeglasses nose-bridge pad has been demonstrated [112]. Different from the contact lens sensors, this tear-sensing platform is placed outside the eye region. The system is enabled by an electrochemical biosensor enclosed within a microfluidic chamber, while the supporting electronics are attached to the inner frame of the eye glasses’ inner frame.

**Figure 16:**
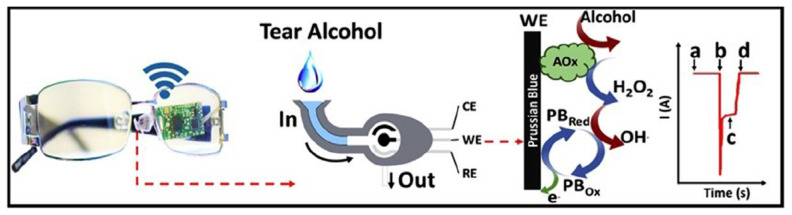
Eyeglasses-based tear sensing system: photo and schematic illustration of the eyeglasses platform consisting of the fluidic device and wireless electronics. A sketch showing enzymatic alcohol detection and signal transduction: (**a**) the baseline of the electrochemical biosensor; (**b**) the current change of the sensor due to the captured tear; (**c**) the measured alcohol signal by the sensor and (**d**) the signal after drying of the sensor. Reproduced from reference [112] with permission from Elsevier.

One example is the alcohol biosensor system (Figure 16). It consists of a fluidic device for collecting the tears, an AO_x_ modified electrochemical alcohol detector, wireless electronics and the supporting eyeglasses platform. For tear collection, a strong capillary force in the inlet enabled by the super-hydrophilic membranes is designed to capture the low volume of tears, leading to the alcohol sensor for detection. Using this system, continuously monitoring the tear alcohol levels of human subjects after alcohol consumption has been carried out. The measurements of three volunteers were successfully validated by comparing to concurrent blood alcohol concentration (BAC) values. It is anticipated that this system can be potentially used to screen and validate the GA biomarkers in tears by replacing the alcohol sensor with GA biomarker sensors.

## 3. Summary and Future Directions

The number of people suffering from neuro-DDs has been rising due to the increasing average age of world population. Hence, sensitive, cost-effective, rapid and reliable diagnostic methods, ideally point-of-care (POC) diagnostic methods for neuro-DDs, are greatly needed. With the discoveries of more and more reliable protein biomarkers in serum, CSF, tear and other biofluids for neuron-DDs, it is becoming increasingly possible that the conventional, expensive and time-consuming diagnostic methods such as neuroimaging or the IOP measurement can be complemented with chip-base detection methods. For instance, the chip-based sensors including contact-lens sensors can serve as point-of-care (POC) equipment for early detection in a rapid and cost-effective manner at home. If the sensors detect the changes of concentrations of the proteins that serve as neuro-DDs’ biomarkers, it is a good indication that further tests are required, making the neuroimaging or eye examination in hospital the next step for the patients’ further diagnosis and treatment. By this way, not only the financial burdens to the patients and the society can be significantly mitigated, but also the frequency of patients’ visiting to hospital and the diagnostic turnaround time can be reduced greatly. More importantly, when taken together, the POC and neuroimaging diagnostics may dramatically improve the diagnostic accuracy of neuro-DDs in a rapid and inexpensive manner.

In this review, some chip-based detection methods for the biomarkers of neuro-DDs have been presented. Until now, not many efforts have been devoted to the chip-based diagnosis of neuro-DDs as evidenced by the limited literatures published in this field (Table 1). Basically, this field is still at its infant stage. Given the many unmet demands of health care from patients with neuro-DDs, it is anticipated that significant research and development efforts will be devoted to this field in the near future. In order to realize POC diagnostics in the future, several challenges still need to be addressed. Undoubtedly, the screening and validation of the most reliable biomarkers in serum, CSF, tears or other biofluids for neuro-DDs in biological and medical research is critical for achieving precise diagnosis and treatment. In terms of technologies, the following aspects of the technology development are equally of great importance. First, given the size of protein biomarkers for neuro-DDs is typically in the range of nanometers, and thus the nanomaterial or nanostructure-enabled sensors are the basis for achieving ultrasensitive detection [113,114,115,116], new nanomaterials and new nanofabrication technologies therefore should be explored for further improving the neuro-DDs detection. Second, for achieving early detection, the sensors need to offer sensitivity and LOD of the neuro-DDs’ biomarkers several orders of magnitude better than diagnostically and physiologically relevant levels with excellent specificity. For POC diagnostics of neuro-DDs, the costs, ease-of-use and portability of the chip-based detection also need to be examined [117,118,119]. It should be noted that the chip-based sensors could be fabricated significantly inexpensive and thus potentially disposable with high-volume production because of the batch-fabrication capability of micro-nano-manufacturing equipment. Most of costs eventually will result from the biomolecules such as antibodies in the bioassay. In this regard, the aptamer is emerging as an ideal candidate to replace the antibody for biodetection, given its low cost, stability and reusability [72,82]. Third, multiplexing capability of the chip-based detection is also of great importance. In other words, the chip-based detection should allow simultaneous detection of a panel of biomarkers of a neurodegenerative disease, which can reduce false positives and thus achieve more reliable detection of the disease. Finally, as a routine procedure, clinic samples need to be processed for the removal of cellular components prior to detection and analysis [120]. The conventional way for sample preparation requires a centrifuge and careful pipetting techniques, which usually requires significant amount of samples and personnel time. In order to take advantages of the chip-based sensing, ideally the sample-preparation functions can be integrated on chip [121,122], thereby minimizing or even eliminating any off-chip steps.

Besides the chip-based detection methods described in this review, it should be noted that some nanoparticles (NPs)-solution based sensors have also been developed and shown great promise for detecting the biomarkers of neuro-DDs [110,123,124,125]. It is anticipated that by integrating the NPs-solution sensors with the chip-based technologies, the sensitivity, LOD and specificity of chip-based detection for neuron-DDs can be further optimized.

## Figures and Tables

**Table 1:** Summary of the chip-based sensing for detecting biomarkers of neurodegenerative diseases (neuro-DDs).

Transducing Mechanism		Biomarkers Detected for Neuro-DDs			Limit-of-Detection (LOD)	Advantages	Limitations
		AD	PD	GA			
Optical	Si/SiO_2_ thin film fluorescence sensor [75]	Aβ42			73.07 pg/mL for Aβ42	High throughput detection; very good sensitivity	Need fluorescent tags to samples; no microfluidic interface; fluorescence microscope is needed for measurement
	Nanoparticle and graphene oxide-enabled SERS [80]	Aβ42, T-tau			100 fg/mL for Aβ42 100 fg/mL for T-tau	No sample preparation; label-free; ultra-sensitivity	Unsuitable for high-throughput detection; SERS testing setup and equipment is needed
	Nanostructures-enabled L-SPR sensor [81]	ADDLs			10 pM for ADDLs	High throughput detection; label-free	Ultraviolet-visible extinction spectroscopy is needed for the test
	SPR [101]		*α*-Syn		<1.3 µM for *α*-Syn	Label-free; possible high throughput detection	SPR testing setup and equipment is needed
	Nanopore thin film sensor [84,98,106]	Aβ42, T-tau	*α*-Syn	IL-12p70	7.8 pg/mL for Aβ42 15.6 pg/mL for T-tau <10 ng/mL for *α*-Syn 2 pg/mL for IL-12p60	High throughput detection; label-free; very good sensitivity	Reflectance spectroscopy is need for the test
Electrical	Interdigitated electrode (IDE)-based sensor [85,100]	Aβ42	*α*-Syn		10 pM for Aβ42 10 pM for *α*-Syn	Label-free; very good sensitivity	No microfluidic interface; Unsuitable for high-throughput detection
	MESFET-based sensor [86]	Aβ42			1 pg/mL for Aβ42	Label-free; very good sensitivity	CNT-MESFET: device-to-device variation and performance non-uniformity
	Single nanopore sensor [94]		*α*-Syn		Not available (NA): single molecule detection for *α*-Syn	Label-free; very good sensitivity	Relative expensive to fabricate single nanopore; need a setup with a patch-clamp amplifier for test
Mechanical	MEMS cantilever sensor [90]		*α*-Syn		<6 ng for *α*-Syn	High throughput detection; label-free; very good sensitivity	Specific testing setup and equipment is needed for monitoring resonance frequency
